# Knowledge about the Developmental Origins of Health and Disease is independently associated with variation in diet quality during pregnancy

**DOI:** 10.1111/mcn.12891

**Published:** 2019-12-12

**Authors:** Luseadra McKerracher, Tina Moffat, Mary Barker, Meghan McConnell, Stephanie A. Atkinson, Beth Murray‐Davis, Sarah D. McDonald, Deborah M. Sloboda

**Affiliations:** ^1^ Department of Anthropology McMaster University Hamilton Ontario Canada; ^2^ Department of Biochemistry and Biomedical Sciences McMaster University Hamilton Ontario Canada; ^3^ MRC Lifecourse Epidemiology Unit University of Southampton Southampton UK; ^4^ Department of Innovation in Medical Education University of Ottawa Ottawa Ontario Canada; ^5^ Department of Anesthesiology and Pain Medicine University of Ottawa Ottawa Ontario Canada; ^6^ Department of Pediatrics McMaster University Hamilton Ontario Canada; ^7^ Department of Obstetrics and Gynecology McMaster University Hamilton Ontario Canada; ^8^ Department of Radiology McMaster University Hamilton Ontario Canada; ^9^ Department of Health Research Health Research Methods, Evidence & Impact McMaster University Hamilton Ontario Canada; ^10^ Farncombe Family Digestive Diseases Research Institute McMaster University Hamilton Ontario Canada

**Keywords:** developmental origins, diet quality, health inequities, knowledge translation, pregnancy, nutrition

## Abstract

Environmental factors affecting development through embryogenesis, pregnancy, and infancy impact health through all subsequent stages of life. Known as the Developmental Origins of Health and Disease (DOHaD) hypothesis, this concept is widely accepted among health and social scientists. However, it is unclear whether DOHaD‐based ideas are reaching the general public and/or influencing behaviour. This study thus investigated whether and under what circumstances pregnant people in Canada are familiar with DOHaD, and if DOHaD familiarity relates to eating behaviour. Survey responses from pregnant people from Hamilton, Canada, were used to assess respondents' knowledge of DOHaD (hereafter, DOHaD_KNOWLEDGE_) compared with their knowledge of more general pregnancy health recommendations (Pregnancy Guideline_KNOWLEDGE_). The survey also characterized respondents' pregnancy diet quality and sociodemographic profiles. We fit two multiple, linear, mixed regression models to the data, one with DOHaD_KNOWLEDGE_ score as the dependent variable and the other with diet quality score as the dependent. In both models, responses were clustered by respondents' neighbourhoods. Complete, internally consistent responses were available for 330 study‐eligible respondents. Relative to Pregnancy Guideline_KNOWLEDGE_, respondents had lower, more variable DOHaD_KNOWLEDGE_ scores. Additionally, higher DOHaD_KNOWLEDGE_ was associated with higher socio‐economic position, older age, and lower parity, independent of Pregnancy Guideline_KNOWLEDGE_. Diet quality during pregnancy was positively associated with DOHaD_KNOWLEDGE_, adjusting for sociodemographic factors. A subset of relatively high socio‐economic position respondents was familiar with DOHaD. Greater familiarity with DOHaD was associated with better pregnancy diet quality, hinting that translating DOHaD knowledge to pregnant people may motivate improved pregnancy nutrition and thus later‐life health for developing babies.

**Key messages**

- It is now well established that early life environmental exposures, especially maternal diet during pregnancy, impact children's development and health across the life span. As such, identification of motivational factors that influence diets during pregnancy is needed.
- Understanding long‐term impacts on children of diet and health during pregnancy may help motivate pregnant people to consume macronutrient and micronutrient dense foods.
- Socio‐economic and related demographic factors are associated with both quality of pregnancy diet and understanding of the long‐term impacts of pregnancy diet.
- To support healthy eating during pregnancy, policy should focus simultaneously on providing income supports for pregnant people unable to afford to prioritize eating high‐quality diets and promoting understanding of the idea that prioritizing high‐quality nutrition during pregnancy has outsized and long‐term effects on children's health.

## 1 INTRODUCTION

Non‐communicable diseases (NCDs) related to obesity and metabolic function (e.g., cardiovascular disease and type 2 diabetes mellitus) represent leading causes of illness and death globally (World Health Organization, 2017). Although these diseases manifest mainly in adulthood, abundant evidence indicates that the main risk factors for metabolism‐related NCDs are established during the peri‐conceptional and foetal stages of development (Barker et al., 2017; Fleming et al., 2018; Stephenson et al., 2018). The idea that early life—especially prenatal—environments shape developmental trajectories, which then affect later‐life disease risk (Barker & Osmond, 1986), is now most widely referred to as the Developmental Origins of Health and Disease (DOHaD) hypothesis (Barnes, Heaton, Coates, & Packer, 2016).

The DOHaD hypothesis offers a powerful set of explanations accounting for much of the epidemiology of NCDs related to metabolic function (Godfrey et al., 2017; Hanson & Gluckman, 2015). Furthermore, models derived from the DOHaD hypothesis indicate that strategies for reducing the burden of metabolic disease should focus on intervening in the earliest stages of life (Garmendia, Corvalan, & Uauy, 2014; Godfrey et al., 2017; Hanson & Gluckman, 2015; Low, Gluckman, & Hanson, 2018; Stephenson et al., 2018; Victora et al., 2016). As such, over the last decade, there has been an increasing push to translate key findings from DOHaD‐related research into social and public health policy and guidelines as well as into clinical practice (Anon., 2011; Barker et al., 2017; Mckerracher, Moffat, Barker, Williams, & Sloboda, 2019).

Indeed, influential stakeholders and institutions in several nations with strong DOHaD‐oriented research communities are beginning to direct efforts towards DOHaD knowledge translation (Baird, Cooper, Margetts, Barker, & Inskip, 2009; Barker et al., 2017; Bay, Mora, Sloboda, & Morton, 2012, 2017b; McMullan et al., 2017; MacNab & Mukisa, 2017; Mckerracher et al., 2019; Oyamada, Lim, Dixon, Wall, & Bay, 2018; Woods‐Townsend et al., 2018; Perreault et al., 2016). What remains unclear is the extent to which DOHaD findings are effectively reaching populations and organizations best positioned to make use of DOHaD‐related research findings. In particular, we do not yet know the extent to which pregnant people and their clinical, social, and local political supports are receiving the idea that early life environments impact metabolic profile and function over the entire lifespan (McMullan et al., 2017). Understanding the current state of DOHaD knowledge translation and its potential impacts is critical to developing next steps, both on the ground and in public policy arenas.

To begin to address this gap in our understanding, this study had three aims: (1) to measure familiarity with key concepts underpinning the DOHaD hypothesis in a sample of pregnant people in Canada, (2) to assess the extent to which familiarity with DOHaD concepts varied within the sample, and (3) to investigate whether having some familiarity with DOHaD concepts might influence behaviour, particularly regarding diet quality during pregnancy.

We hypothesized that, because DOHaD knowledge translation efforts are relatively new in general and in Canada in particular (Mckerracher et al., 2019), people outside of the research community would be largely unfamiliar with DOHaD‐related concepts. We predicted that pregnant Canadians would have lower familiarity with DOHaD‐related concepts relative to their familiarity with general pregnancy health guidelines as issued by Health Canada. The core of these latter guidelines has been in place since 1995 (Haertsch, Campbell, & Sanson‐Fisher, 1999) and is widely communicated by public health practitioners and media channels (Chalmers, Dzakpasu, Heaman, & Kaczorowski, 2008).

We further hypothesized that pregnant people's understanding of DOHaD‐related concepts would vary and that this variation would be patterned sociodemographically. Health knowledge and level of trust in health workers and systems differs substantially among communities and sociodemographic groups in Canada (Glouberman & Millar, 2003; Jenkins & Keating, 1999), as elsewhere (Chinn, 2011; Sørensen et al., 2012). Specifically, people of higher socio‐economic position (SEP; i.e., higher income, greater material wealth, and more years of formal education) tend to be more knowledgeable about their own health and more trusting of health care institutions (Sørensen et al., 2015; Willems, De Maesschalck, Deveugele, Derese, & De Maeseneer, 2005). As such, these people generally have greater health literacy (ability to understand health information and to navigate health systems) and a stronger sense of self‐efficacy in relation to health care (Nutbeam, 2008; Smith, Dixon, Trevena, Nutbeam, & McCaffery, 2009). Additionally, older people with greater parenting experience have had more opportunities to accrue knowledge and understanding (Bornstein, Cote, Haynes, Hahn, & Park, 2010). Multi‐year experience with navigating and understanding a given health system is also associated with improved health knowledge and literacy (Kalich, Heinemann, & Ghahari, 2016; Simich, 2009). Thus, we anticipated that familiarity with DOHaD concepts would be positively associated with respondents' SEP, age, and parity and negatively associated with respondents' statuses as newcomers to Canada, holding constant familiarity with general pregnancy health guidelines.

The last hypothesis we tested was that pregnant respondents would be more likely to prioritize eating nutritious foods if they understood that what they ate during pregnancy could impact their children's health not only perinatally but through subsequent stages of life. Our test prediction was that self‐reported quality of diet during pregnancy would be positively associated with familiarity with DOHaD‐related concepts, independent of other factors known/expected to influence pregnancy diet (age, SEP, parity, newcomer status, and knowledge of general pregnancy health recommendations).

## 2 MATERIALS AND METHODS

### 2.1 Setting and participants

Participation in this study was restricted to people who were pregnant and living in Hamilton, Canada, at the time of data collection (between December 2015 and September 2018). Hamilton is a suitable site to evaluate our hypotheses for three main reasons. First, it is sociodemographically diverse, with known, marked variation in access to knowledge, support, and financial resources (Dean & Elliott, 2012; DeLuca, Buist, & Johnston, 2012; Latham & Moffat, 2007; Moffat & Galloway, 2008). Second, the city has a high prevalence of obesity and metabolism‐related NCDs for an urban centre of its size (Navaneelan & Janz, 2014). Lastly, Hamilton is characterized by well‐documented, systematic health inequities among neighbourhoods, including in early life indicators of later‐life NCD risks such as low birthweight (DeLuca et al., 2012).

We advertised our anonymous survey, described further below, at community health centres, midwifery clinics, prenatal classes, hospitals, and other community organizations across Hamilton. We used a targeted approach when advertising our survey to ensure that pregnant people from diverse backgrounds were appropriately represented in our sample. That is, we promoted the study intensively at community health centres with prenatal programming where attendees were relatively likely to come from low‐income, low‐educational attainment households (Denny & Grady, 2007) and/or to identify as non‐White and/or as newcomers to Canada (Durant et al., 2007; Oh et al., 2015).

### 2.2 Study instrument

We developed on‐line (LimeSurvey©) and print versions of a 140‐item questionnaire in English. It was piloted with 78 pregnant people between December of 2015 and August of 2016 to ensure reliability of items and range of response selection across items. It was slightly modified, then relaunched, and rerun between June 2017 and September 2018; following the relaunch, a print version was also translated and made available in Arabic, the most common language spoken at a main study promotion location. The questionnaire included questions grouped into the following four domains: (1) the extent to which respondents were familiar with core concepts and evidence related to the DOHaD hypothesis (hereafter, DOHaD_KNOWLEDGE_), (2) the extent to which respondents were familiar and in agreement with Health Canada's general guidelines for health during pregnancy (hereafter, Pregnancy Guideline_KNOWLEDGE_), (3) respondents' self‐reported diet quality during pregnancy, and (4) respondents' sociodemographic characteristics.

### 2.3 Measures

To assess DOHaD_KNOWLEDGE_, we aggregated scores concerning the level at which respondents agreed, using a 5‐point Likert scale (0 = *strongly disagree* to 4 = *strongly agree*), with each of five statements about the long‐term impacts of peri‐conceptional and prenatal and perinatal parental and/or grandparental health and behaviour on children's health. The five statements are presented in Table 1. The aggregated scores resulted in a 20‐point DOHaD_KNOWLEDGE_ scale, which could take a minimum value of 0 (indicating no knowledge/familiarity of concepts and evidence related to the DOHaD hypothesis) and a maximum value of 20 (indicating very strong knowledge and uptake of such concepts and evidence).

**Table 1:** Components of the DOHaD_KNOWLEDGE_ scale and the Pregnancy Guideline_KNOWLEDGE_ scale

DOHaD_KNOWLEDGE_ scale	What a woman eats during her pregnancy affects her baby's risk of becoming obese as an adult.
	What a woman eats during her pregnancy affects her grandchildren's risk of becoming obese.
	Before pregnancy, both what the mother and the father eat affects the growth and health of their baby.
	What I ate before pregnancy affects my child's chance of becoming obese as an adult.
	What I eat while I am breastfeeding affects my child's chance of becoming obese as an adult.
Pregnancy Guideline_KNOWLEDGE_ scale	Smoking during pregnancy will harm my baby.
	Taking daily prenatal vitamins during my pregnancy is good for my baby's health.
	Pregnant women should not eat as much as they like because they are “eating for two.”
	Eating nutritious food is my top priority.
	I would consider ____ to be a healthy birthweight for my baby. (Options: ≤1.5 kg = 0 points; 1.5–2.5 kg = 1 point; 2.5–3 kg = 2 points; 3–3.5 kg = 4 points; 3.5–4 kg = 5 points; 4–4.5 kg = 2 points; ≥4.5 kg = 0 points)

Pregnancy Guideline_KNOWLEDGE_ was assessed in the same way as DOHaD_KNOWLEDGE_, but using three statements central to Health Canada's general recommendations for having a healthy pregnancy, one statement implicit in Health Canada's recommendations, and one statement based on its prenatal guidelines for health professionals. These Pregnancy Guideline_KNOWLEDGE_ statements are presented in Table 1, alongside those comprising DOHaD_KNOWLEDGE_. The first four of these Pregnancy Guideline_KNOWLEDGE_ statements were assessed on a 5‐point Likert scale. The last was assessed on a 5‐point scale based on how close a participant's selected response came to Health Canada's reported 50th percentile for a healthy, full‐term birthweight as of 2016 (3.5 kg). As with DOHaD_KNOWLEDGE_, Pregnancy Guideline_KNOWLEDGE_ statements were aggregated into a 20‐point scale, ranging from 0 to 20. To further probe respondents' familiarity with Health Canada's pregnancy guidelines, respondents were also given the opportunity to answer two open‐ended questions concerning foods to eat and foods to avoid during pregnancy. These responses were not included in the Pregnancy Guideline_KNOWLEDGE_ scale but are reported briefly in Text S1.

Dietary practices during pregnancy were reported by the respondents using the PrimeScreen© Food Frequency Questionnaire (Rifas‐Shiman et al., 2001), which was integrated into our larger questionnaire. Respondents were asked to record how often during their pregnancies they ate foods from each of 20 mutually exclusive food categories. Possible responses ranged from “never” to “more than twice a day.” Frequent consumption of foods from each nutrient‐dense food category was given a positive score up to a maximum of 4 (with the exception of the category low‐fat dairy foods, which could take a maximum value of 8). Frequent consumption of each nutrient‐poor food was given a negative score, with the most negative possible value being −4. All possible responses to the food frequency questions are presented in Table 2, along with three example food categories and their associated scores. A full list of food categories and scores is available in Text S2 and Table S1. After scoring each individual food category, a total of all food category scores for each respondent was calculated to give a diet quality score, which could take a minimum value of −16 and a maximum of 36.

**Table 2:** PrimeScreen© Food Frequency Questionnaire (Rifas‐Shiman et al., 2001)

Example food category	More than once a day	Almost daily	Two to four times a week	Once a week	Less than once a week	Never
Dark green leafy vegetables (e.g., kale, turnip greens, bokchoy, and Swiss chard)	□ +4	□ +3	□ +2	□ +1	□ 0	□ 0
Fish/seafood	□ +4	□ +3	□ +2	□ +1	□ 0	□ 0
Sugary drinks (e.g., soda, fruit drinks, and Gatorade)	□ −4	□ −3	□ −2	□ −1	□0	□ 0

The sociodemographic characteristics assessed in the survey comprised respondent's age (years), highest completed level of education (ranging from 1 = *had not obtained a high school diploma* to 4 = *had completed post‐secondary degree*; further information on this variable available in Text S3 and Table S2), household annual income bracket (1 = <$23,000, 2 = $23,000–$39,999, 3 = $40,000–$79,999, and 4 = >$80,000), main source of household income (wages/salaries; other, e.g., social assistance and inheritance), current postal (zip) code, self‐identified ethnicity, status as a newcomer or not (temporary residents and permanent residents of Canada who had lived in Canada ≤5 years vs. Canadian citizens and permanent residents who had lived in Canada >5 years), marital status (married/common‐law partnership, single, divorced, widowed, and others), and parity. Details on collation of these sociodemographic variables are reported in Section 2.4

### 2.4 Data preparation

We began by exploring the data, characterizing the sample sociodemographically, reducing the number of sociodemographic predictors (to avoid multicollinearity and model overfitting in regression analyses, described below), and removing 63 cases for which participants discontinued responding within the first 20 survey questions. We also removed one duplicate submission, six submissions for which respondents' postal codes fell outside of Hamilton's boundaries, and four submissions in which there were clear internal inconsistencies in responses.

To decrease the number of sociodemographic variables included in our models, we combined those directly related to SEP (respondent's household income bracket, respondent's educational attainment group, and source of income) into an SEP index (hereafter, SEP score) for use in statistical analyses. SEP, an additive score of educational and income brackets, ranges from two to eight. A score of 2 indicates that, at the time of data collection, the respondent had not completed high school AND lived in a household in which the annual income is <$23,000; all such respondents also indicated some form of social assistance as a source of income. A score of 8 indicates that the respondent had completed at least one college/university degree AND lived in a household with an annual, employment‐based income >$80,000. A detailed breakdown of all SEP scores is available in Text S3 and Table S3.

Other intercorrelated sociodemographic variables did not lend themselves to straightforward amalgamation, so we selectively dropped out those that were highly collinear with others but for which we did not have theoretical justifications for retaining in our models. Specifically, marital status and self‐reported non‐White ethnicity were not included because we had not identified them a priori as independent drivers of pregnancy health knowledge or pregnancy diet quality, and they were correlated with respondents' ages, SEP, parities, and/or with status as newcomers to Canada. Further details on the relationships among sociodemographic variables, along with the results of one sensitivity model in which we adjusted for non‐White ethnicity instead of newcomer status, are available in Text S3.

Postal code, truncated to its first three digits to represent 25 neighbourhood areas in Hamilton, was treated as a clustering variable, because previous empirical research indicates that sociodemographic variables, behavioural patterns, and health outcomes are strongly geographically patterned in the city (DeLuca et al., 2012). So, treating respondents from the same neighbourhoods as observations independent from one another is inappropriate.

### 2.5 Statistical analyses

We carried out four sets of analyses, the first to assess the internal reliability of our DOHaD_KNOWLEDGE_ and Pregnancy Guideline_KNOWLEDGE_ scales and the subsequent ones to evaluate our three test predictions.

To assess the internal consistency of the DOHaD_KNOWLEDGE_ and Pregnancy Guideline_KNOWLEDGE_ scores, we calculated the Cronbach's α (Cronbach & Murphy, 1970) for each scale. This statistic can take values between 0 and 1. A Cronbach's α above ~.7 indicates that component scale items take similar values for a given respondent, whereas totalled scores vary substantially among participants' responses. Low Cronbach's α values (below .5) may indicate either low intra‐item cohesiveness within respondents or low inter‐respondent variation in responses.

To evaluate our first prediction that DOHaD_KNOWLEDGE_ is less likely to have successfully reached pregnant people than Pregnancy Guideline_KNOWLEDGE_, we compared means (using a Wilcoxon ranked sum test) and coefficients of variation between the two knowledge scales. To assess our second prediction that DOHaD_KNOWLEDGE_ is likely to be sociodemographically patterned, we fit a linear mixed effects regression model using maximum likelihood to the data. Fixed effects in the model were respondent's age, SEP score, status as a newcomer to Canada, and parity. Intercepts were allowed to vary randomly among the 25 postal code (neighbourhood) groups. As SEP score is strongly left skewed, resulting in heteroscedasticity in the model's residuals, we power‐transformed this variable for use in a sensitivity analysis, raising the exponent to five, at which point the residual error appears homoscedastic (Text S4 and Figure S1). Third, to assess the prediction that DOHaD_KNOWLEDGE_ would be associated with greater positive attention to health‐related behaviours during pregnancy, we modelled diet quality score as a maximum likelihood function of DOHaD_KNOWLEDGE_, adjusting for respondent's age, SEP score, newcomer status, Pregnancy Guideline_KNOWLEDGE_ score and parity, again clustering the data by postal code group and allowing intercepts to vary randomly among neighbourhoods. No data transformations or other modifications to this model appeared necessary (Text S4 and Figure S2).

Alpha was set at *P* = 0.05 for all statistical analyses. All analyses were carried out and all plots were made in the stat (R Core Development Team, 2017), nlme (Pinheiro, Bates, DebRoy, Sarkar, & Team, 2018), psych (Revelle, 2018), and ggplot2 (Wickham, 2016) packages of the statistical environment, R.

### 2.6 Research ethics approval

The study protocol used to evaluate these predictions was reviewed and approved by the Hamilton Integrated Research Ethics Board, approval #0570.

## 3 RESULTS

**Figure 1:**
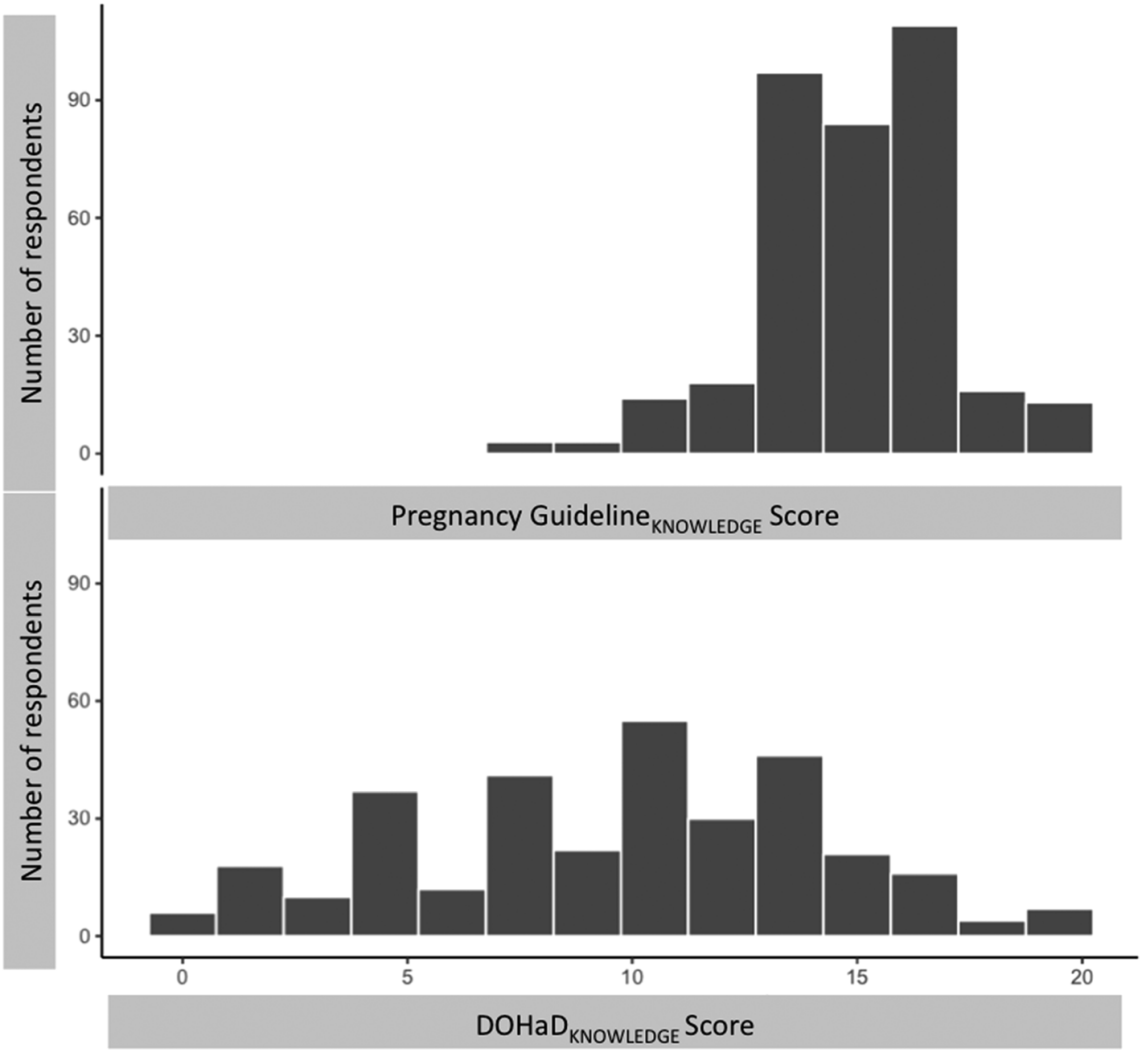
Frequency of responses from participants in each socio‐economic position (SEP) bracket. SEP scores of 2–4 indicate lower household income (<$23,000 per year) and low (no completed post‐secondary) respondent educational attainment levels. SEP scores of 5–7 indicate higher respondent educational attainment levels (at least some post‐secondary) and household income brackets near the city's median ($40,000–$79,000). SEP scores of 8 indicate high respondent educational attainment levels (completion of at least one post‐secondary degree) and household incomes >$80,000 per year. Seventy (21%) of respondents have SEP scores of ≤4, 127 (38%) have scores of 5–7, and 133 (40%) have SEP scores of 8.

Complete, internally consistent responses to all questions used to assess DOHaD_KNOWLEDGE_, Pregnancy Guideline_KNOWLEDGE_, diet quality, respondent's age, SEP score, parity, and status as a newcomer to Canada were available from 330 respondents, inclusive of the 78 respondents to the pilot. Descriptive statistics for the continuous sociodemographic characteristics, DOHaD_KNOWLEDGE_ score, Pregnancy Guideline_KNOWLEDGE_ score, and diet quality score are presented in the text below and in Table 3. The distribution of SEP scores is also represented in Figure 1.

**Table 3:** Descriptive statistics for continuous and ordinal sociodemographic characteristics of sample and for DOHaD_KNOWLEDGE_ score, Pregnancy Guideline_KNOWLEDGE_ score, and diet quality score: *N* = 330

Characteristic	Min	Median (IQR)	Mean (*SE*)	Max
DOHaD_KNOWLEDGE_ score	0	10 (6–13)	9.4 (±0.25)	20
Pregnancy Guideline_KNOWLEDGE_ score	7	15 (14–16)	14.5 (±0.10)	20
Diet quality score	−14	12 (5–18)	11.4 (±0.52)	36
Maternal age (years)	17	30 (27–34)	30.5 (±0.29)	47
SEP score	2	7 (5–8)	6.4 (±0.11)	8
Number of previous births	0	0 (0–1)	0.8 (±0.06)	6
Status as newcomer to Canada (0 = *has lived in Canada more than 5 years* and 1 = *has lived in Canada fewer than 5 years*)	0	N/A	N/A	1

Participants varied sociodemographically, ranging in age from 17 to 47 (mean = 30.4, *SE* = ±0.29), in SEP score from two to eight (mean = 6.4, median = 7, *SE* = ±0.11), and in parity from zero to six (mean = 0.8, *SE* = ±0.06). Nine percent of respondents were identified as newcomers to Canada (i.e., they had immigrated to Canada within the 5 years before participating in the study).

The DOHaD_KNOWLEDGE_ scale was internally reliable, with a Cronbach's α of .82, indicating that its component statements effectively measure the same mental construct (Cronbach & Murphy, 1970). Respondents' DOHaD_KNOWLEDGE_ scores ranged from 0 to 20, with a mean (*SE*) of 9.4 (±0.25; Table 3).

Pregnancy Guideline_KNOWLEDGE_ scale had a low Cronbach's α (.27). This low value can be attributed to low inter‐individual variation in responses to any items (90% of respondents scored 4 or 5 on all five items), rather than to low inter‐item agreement. Respondents' pregnancy Guideline_KNOWLEDGE_ scores took values ranging from 7 to 20. The mean (*SE*) of this variable was 14.5 (±0.10; Table 3).

The minimum diet quality score was −14 and the maximum was 36. The mean (*SE*) for this variable was 11.4 (±0.52).

### 3.1 Analysis for Hypothesis 1 (comparing DOHaD_KNOWLEDGE_ and Pregnancy Guideline_KNOWLEDGE_ scores)

**Figure 2:**
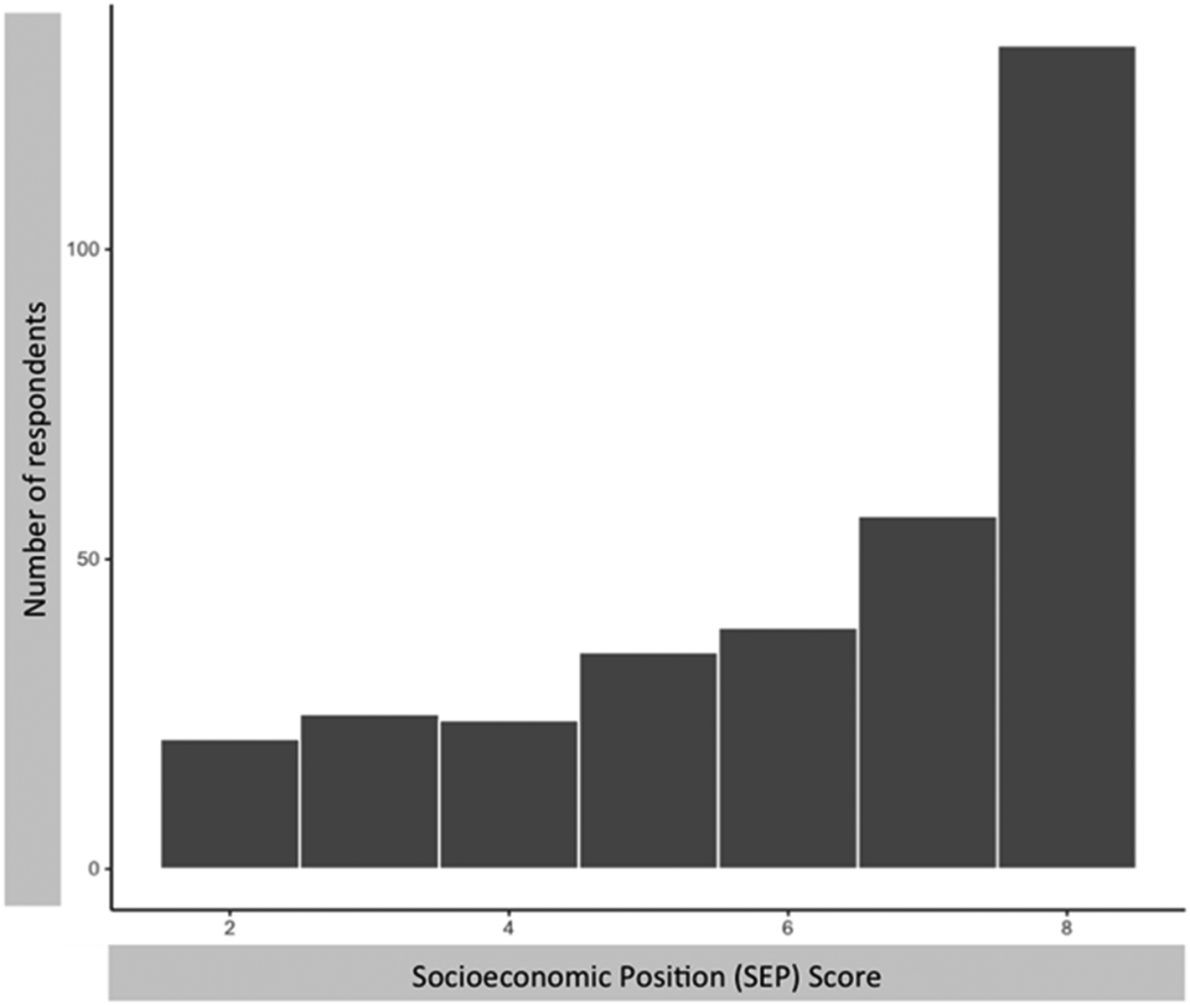
Distributions of respondents' Pregnancy Guideline_KNOWLEDGE_ and DOHaD_KNOWLEDGE_ scores. Ninety‐one (27%) of respondents have Pregnancy Guideline_KNOWLEDGE_ scores of 16, the modal score for this variable. Forty (12%) of respondents have DOHaD_KNOWLEDGE_ scores of 10, the modal score for this variable

The mean DOHaD_KNOWLEDGE_ score of 9.4 was ~5 points lower than the sample's mean Pregnancy Guideline_KNOWLEDGE_ score of 14.5 (*P* = 0.000). The coefficient of variation for DOHaD_KNOWLEDGE_ score was 0.49, indicating substantial variation in respondents' familiarity with DOHaD‐related concepts. The coefficient of variation for Pregnancy Guideline_KNOWLEDGE_ was 0.13, indicating a high level of consensus among respondents in whether to agree with the statements this scale comprises. These differences in the spreads and central tendencies between DOHaD_KNOWLEDGE_ and Pregnancy Guideline_KNOWLEDGE_ are summarized in Figure 2. Moreover, respondents' qualitative answers about foods to eat and to avoid during pregnancy aligned with recommendations from Health Canada (Text S1), further supporting a high level of consensus with respect to Pregnancy Guideline_KNOWLEDGE_.

### 3.2 Analysis for Hypothesis 2 (inequities in DOHaD_KNOWLEDGE_)

**Figure 3:**
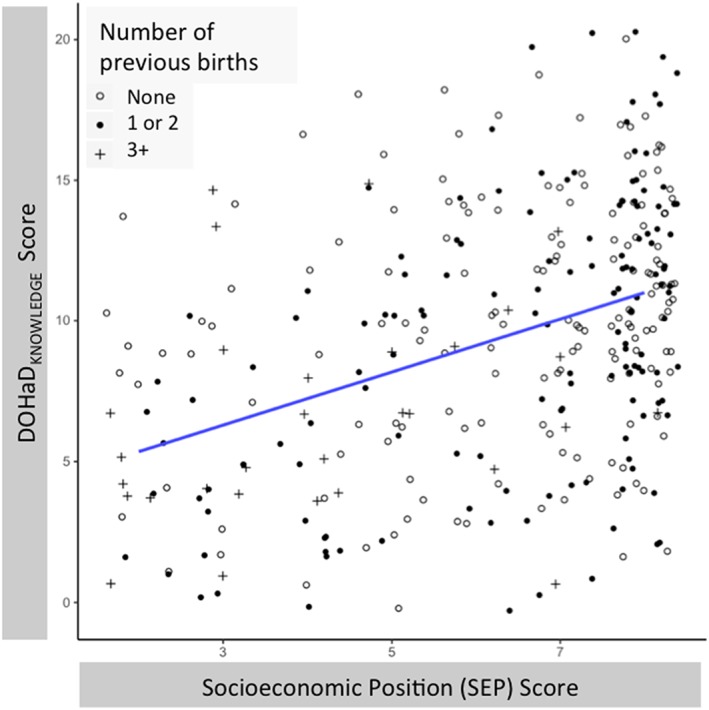
Scatterplot of linear relationship between DOHaD_KNOWLEDGE_ score and SEP score. Data are presented as individual scores of DOHaD_KNOWLEDGE_ and SEP. Open circles = respondents without prior pregnancies, closed circles = respondents who have one or two children, and plusses = respondents with three and more children. As SEP score increases, DOHaD_KNOWLEDGE_ score increases. Respondents with three and more children (plusses) are relatively likely to fall below the fit line.

The results of the DOHaD_KNOWLEDGE_ by sociodemographic factors regression are presented in Table 4. Consistent with our predictions, we found that DOHaD_KNOWLEDGE_ was positively associated with SEP score (Figure 3) and respondent's age, adjusting for status as a newcomer to Canada, Pregnancy Guideline_KNOWLEDGE_ score, and parity and clustering the data by neighbourhood. There was no clear statistical evidence that DOHaD_KNOWLEDGE_ is independently associated with status as a newcomer to Canada or with Pregnancy Guideline_KNOWLEDGE_. DOHaD_KNOWLEDGE_ was negatively associated with parity, adjusting for all other predictors.

**Table 4:** Relationships between DOHaD_KNOWLEDGE_ score and maternal age, SEP score, previous childbearing experience, and Pregnancy Guideline_KNOWLEDGE_ score in a full, linear mixed effects model

Fixed effect	Estimate	95% CI	*P* value
SEP score	0.78	[0.49, 1.07]	0.000***
Maternal age (years)	0.12	[0.02, 0.21]	0.021*
Number of previous births	−0.47	[−0.91, −0.02]	0.040*
Status as a newcomer to Canada	1.44	[−0.05, 3.03]	0.078†
Pregnancy Guideline_KNOWLEDGE_	0.22	[−0.01, 0.45]	0.062†

*Note*. Intercepts were allowed to vary randomly among postal code groupings (neighbourhoods). Model summary: AIC = 1892.8, log likelihood = −938.4.

* *P* ≤ 0.050.

** *P* ≤ 0.010.

*** *P* ≤ 0.001.

† *P* ≤ 0.100.

Although we report here the results of the model using raw SEP score as a predictor to simplify interpretation of this analysis focused largely on SEP, we acknowledge that the error is likely mis‐specified in this model, as the residual plot indicates high levels of heteroscedasticity (Text S4 and Figure S1a). Transforming SEP score to the fifth exponent eliminates this problem (Text S4 and Figure S1b), and the model in which (SEP score)^5^ is included in lieu of raw SEP score produces results consistent with our main findings (see Text S4 and Table S4).

### 3.3 Analysis for Hypothesis 3 (factors associated with diet quality score)

**Figure 4:**
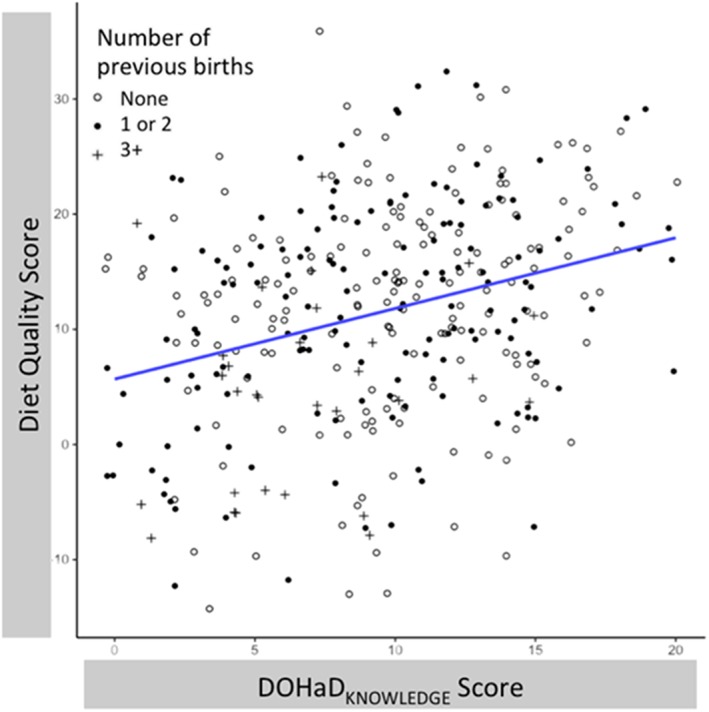
Scatterplot of linear relationship between diet quality score and DOHaD_KNOWLEDGE_ score. Data are presented as individual scores of diet quality score and DOHaD_KNOWLEDGE_ score. Open circles = respondents without prior pregnancies, closed circles = respondents who have one or two children, and plusses = respondents with three and more children. As DOHaD_KNOWLEDGE_ score increases, diet quality score increases. Respondents with three and more previous children (plusses) are relatively likely to fall below the fit line.

The results of the analysis in which we regressed diet quality score on DOHaD_KNOWLEDGE_ score, Pregnancy Guideline_KNOWLEDGE_ score, and sociodemographic factors are summarized in Table 5. Consistent with expectations, diet quality score was positively associated with DOHaD_KNOWLEDGE_ score (Figure 4). Diet quality score was also positively associated with respondent's age, SEP score, and negatively associated with parity. We highlight also that there was no statistical evidence that diet quality score was independently associated with general Pregnancy Guideline_KNOWLEDGE_ score.

**Table 5:** Relationships between diet quality score and DOHaD_KNOWLEDGE_ score, maternal age, SEP score, previous childbearing experience, and Pregnancy Guideline_KNOWLEDGE_ score in a full, linear mixed effects model

Fixed effect	Estimate	95% CI	*P* value
DOHaD_KNOWLEDGE_ score	0.35	[0.13, 0.56]	0.003**
SEP score	0.62	[0.00, 1.24]	0.051^‐^
Age	0.31	[0.10, 0.51]	0.003**
Number of previous births	−1.35	[−2.27, −0.43]	0.005**
Status as a newcomer to Canada	−6.46	[−3.17, 1.12]	0.062†
Pregnancy Guideline_KNOWLEDGE_	0.04	[−0.44, 0.52]	0.869

*Note*. Intercepts were allowed to vary randomly among postal code groupings (neighbourhoods). Model summary: AIC = 2372.0, log likelihood = −1177.0.

* *P* ≤ 0.050.

** *P* ≤ 0.010.

*** *P* ≤ 0.001.

† *P* ≤ 0.100.

## 4 DISCUSSION

We developed and used a novel instrument to show that most respondents were very knowledgeable about general pregnancy health recommendations as laid out by Health Canada (Pregnancy Guideline_KNOWLEDGE_), but strikingly few respondents, by comparison, were knowledgeable about DOHaD concepts (DOHaD_KNOWLEDGE_). Further, the data indicate that DOHaD_KNOWLEDGE_ is inequitably distributed with higher DOHaD_KNOWLEDGE_ scores concentrated among respondents of higher SEP, older age, and lower parity. We also show that those respondents who have some understanding of DOHaD were more likely to report a more nutrient‐dense diet during pregnancy, above and beyond what well‐known diet quality predictors like age, parity, and SEP would predict. We argue that these inequities in DOHaD_KNOWLEDGE_ and in pregnancy diet quality may be partly attributable to politically‐, institutionally‐, and historically structured differences among sociodemographic subgroups within the sample in the dissemination, accessibility, and/or uptake of DOHaD concepts.

### 4.1 Familiarity with pregnancy health guidelines, but not their long‐term implications

Although respondents were clearly familiar with general recommendations about how to live healthfully during pregnancy, they were generally unfamiliar with the idea that prenatal health/nutrition influences the long‐term obesity and metabolic disease risks of their children. This may suggest that, as predicted, pregnancy guidelines are being systematically and confidently disseminated and monitored by reputable sources, mainly Health Canada (Chalmers et al., 2008). While Health Canada is educating health professionals about the long‐term consequences of excessive gestational weight gain (Health Canada, 2014), it may not yet be systematically disseminating the idea directly to pregnant people that the early life nutritional environment plays a significant role in determining later‐life obesity and metabolic disease risks.

Although these results align with our test predictions regarding knowledge translation patterns, we acknowledge that there are other plausible explanations for the discrepancy in scores between the DOHaD_KNOWLEDGE_ and Pregnancy Guideline_KNOWLEDGE_ scales. One such alternative explanation includes how cognitively easy or difficult it is to assimilate concepts measured by each of the scales. The DOHaD hypothesis has been discussed academically for the last 30 years (Barnes et al., 2016), but its translation has been difficult (McKerracher et al., 2019). The core idea that the early life environment shapes how you grow and thus can affect your later‐life health is relatively straightforward. However, what exactly is meant by environment, how different kinds of environments might affect growth and development and in what ways, and how developmental trajectories might influence risks for developing diabetes, obesity, or heart disease decades after exposures are not easily understandable (Barnes et al., 2016; Winett, Wallack, Richardson, Boone‐Heinonen, & Messer, 2016, Winett, Wulf, & Wallack, 2016). Furthermore, the DOHaD hypothesis confronts the dominant Western narrative about obesity and metabolic health (Monaghan, 2017; Warin, Turner, Moore, & Davies, 2008). Namely, Westerners most often intuit that individuals, especially adults, are responsible for and cause their own health‐related behaviours and health outcomes (Marmot & Bell, 2019; Winett, Wallack, et al., 2016). The DOHaD hypothesis shifts responsibility for health outcomes away from individuals to the multiple layers of the environment in which we developed. These environmental layers include our mothers' bodies, our familial histories, our household economies, our communities, and the political structures in which our communities are embedded (Winett, Wallack, et al., 2016; Winett, Wulf, & Wallack, 2016). Whereas Health Canada's general pregnancy nutrition guidelines are compatible with a cognitively easy, traditional narrative, arguably, the DOHaD concepts are not.

Regardless of which proximate barriers prevent understanding of DOHaD ideas, these data indicate that pregnant people in Hamilton are well informed about what the general dietary and behavioural guidelines for pregnancy health are. But, they do not necessarily understand why they should eat in a particular way, or what the long‐term consequences of their prenatal health might be for their children's health.

### 4.2 Inequity in who has DOHaD knowledge

Our initial hypothesis that inequities in health literacy are likely to drive inequities in familiarity with DOHaD‐related concepts offers a compelling explanation for our findings that DOHaD_KNOWLEDGE_ score varies with SEP and age. This is consistent with the handful of previous studies that have assessed DOHaD_KNOWLEDGE_ translation, which show that people living in lower SEP contexts tend to have lower health knowledge and literacy generally, have little self‐efficacy with which to make use of health knowledge, and are more distrustful of health service provision (Barker et al., 2017; Sørensen et al., 2015). Fortunately, the available literature also suggests that these kinds of barriers can be overcome. DOHaD‐related concepts have been successfully translated to several hard‐to‐reach, albeit selected, populations with low levels of health literacy (adolescents in New Zealand and the Cook Islands, adolescents in Uganda, and low SEP women in the United Kingdom; Baird et al., 2014; Bay et al., 2012, 2016a, Bay, Morton, & Vickers, 2016b, Bay, Vickers, Mora, SLoboda, & Morton, 2017a, Bay et al., 2017b; MacNab & Mukisa, 2017; Barker et al., 2017). In these selected cohorts, knowledge translation has required development of hands‐on curricula and/or deep engagement and cultivation of meaningful relationships with community partners but is proving fruitful (Grace & Bay, 2011; MacNab & Mukisa, 2017).

The data reported here indicate that DOHaD_KNOWLEDGE_ score is negatively associated with respondents' parity. This is counter to our initial expectations, where we had assumed more pregnancy “experience” would lead to more knowledge. However, this negative association accords with some data from other populations indicating that experienced mothers are less likely to seek out information about prenatal health than first time mothers, relying instead on their own past experiences (Declercq, Sakala, Corry, & Applebaum, 2007). Equally, evidence from other populations suggests that health service providers reduce efforts to engage with pregnant people who they perceive to be “experienced” (Blondin & LoGiudice, 2018; Downs, Savage, & Rauff, 2014). As such, pregnant people who have previously given birth may have had and/or taken fewer opportunities to access and assimilate information about DOHaD.

### 4.3 Why pregnancy diet quality might be independently associated with DOHaD knowledge

The significant independent association between pregnancy diet quality and an understanding of DOHaD concepts does not imply a causal relationship between DOHaD_KNOWLEDGE_ and diet quality score, but it might hint at one. It is well established that motivation is central to engaging in positive health behaviours like eating healthfully during pregnancy (Martin, Haskard‐Zolnierek, & DiMatteo, 2010), and health behaviours and health surveillance are more salient to pregnant people than to the general population (Phelan, 2010). Additionally, understanding the relevance of a set of recommendations may contribute to motivating people to follow them (Deci & Ryan, 2002). However, because numerous other factors were also associated with variation in pregnancy diet quality such as age, parity (Table 4), and main source of household income (data not shown), these other factors likely constrain and/or mediate the relationship between diet quality and DOHaD_KNOWLEDGE_. Nonetheless, if ongoing qualitative research in the study population provides evidence further supporting hypothesized links between deeper understanding, motivation, and eating behaviour, continuing efforts at DOHaD knowledge translation may pay off in improved diets during pregnancy and, ultimately, better health outcomes and reduced health inequities for mothers and babies.

### 4.4 Limitations

We recognize that our study has some limitations that should be considered going forward. Specifically, the sample is biased towards higher SEP respondents (Figure 1). Despite efforts to target hard‐to‐reach, marginalized segments of Hamilton's population, our highest rates of response came from relatively educated, affluent pregnant Hamiltonians. Nonetheless, 24% of respondents did come from the lowest income bracket. Additionally, responses to other parts of the survey's sociodemographic section as well as our own field observations suggest that we were able to sample not only lower SEP Hamiltonians but also those who identify as Indigenous, as newcomers to Canada, and as racialized minorities, suggesting our analyses reflect much of the city's sociodemographic diversity. Moreover, although caution is necessary, we highlight that, in spite of the sample's noisiness, we found clear signals indicating that we should reject the null hypotheses of no relationships between DOHaD_KNOWLEDGE_ score, diet quality score, and sociodemographic factors.

We also recognize the need for validated instruments to measure DOHaD_KNOWLEDGE_ and Pregnancy Health Guideline_KNOWLEDGE_. Specifically, both scales comprise predominantly positively phrased statements, and we should aim for an equal number of positively and negatively phrased items in each scale. Our team is thus working towards further developing and validating these scales. At the same time, though, the high internal reliability of the DOHaD_KNOWLEDGE_ scale suggests that a coherent mental construct is being effectively measured (Gliem & Gliem, 2003). We also note that, with the exception of recent work by Oyamada and colleagues in Japan (2018), we are the first to measure DOHaD_KNOWLEDGE_ using multiple items. Our method builds on previous work, which has used a single question/statement to assess familiarity with DOHaD (e.g., Bay et al., 2012, 2017b); it thus represents an important step towards better characterizing how people think about DOHaD‐related ideas.

Lastly, as mentioned, earlier work emphasizes that effective DOHaD knowledge translation depends on deep understandings of the needs and priorities of communities and genuine efforts at relationship‐building and mutual exchange (Baird et al., 2014; Barker et al., 2017; Bay et al., 2012; Lawrence et al., 2016; MacNab & Mukisa, 2017). Obtaining a deep understanding of pregnancy health and nutrition experiences solely through an anonymous questionnaire is impossible. As such, the survey used here constitutes only one prong of a research strategy involving engagement with a wide variety of community stakeholders in Hamilton through focus group discussions, stakeholder meetings, and participant observation. In future work, we will situate survey responses in a tapestry of contextual information. Until then, these anonymous data highlight some gaps in respondents' understanding and therefore suggest where and how we might focus our attention when translating knowledge via stakeholder engagement.

## 5 CONCLUSIONS

We investigated familiarity of pregnant respondents living in Hamilton, Canada, with concepts related to the DOHaD hypothesis. There were substantial inequities in access to and/or uptake of DOHaD concepts. Furthermore, evidence indicated that having more familiarity with DOHaD‐related concepts is associated with relatively high‐quality pregnancy diet, suggesting that inequities in DOHaD knowledge may be associated with inequities in a health‐related behaviour during a window critical for the development and long‐term health of children to be. These findings underscore the potential value of DOHaD knowledge translation efforts. Moreover, deep collaboration with community and political partners can build capacity for supporting pregnancy health and nutrition not only now, but for the generations to come.

## CONFLICTS OF INTEREST

The authors declare that they have no conflicts of interest.

## CONTRIBUTIONS

All authors contributed to study design, study promotion/data collection, interpretation of results, and editing and approval of manuscript; LM collated and analysed data and drafted majority of manuscript text; TM, MB, and DS drafted portions of manuscript text.

## Supporting information


**Figure S1:** Comparison of plots of residual error versus fitted values between analysis 2 model using raw SEP score (DOHaDKNOWLEDGE score~raw SEP score + maternal age + newcomer status + number of previous births) and sensitivity model using transformed SEP score (DOHaDKNOWLEDGE score~ (SEP score)5 + maternal age + newcomer status + number of previous births).
**Figure S2:** Residual error versus fitted values from Diet Quality score~ DOHaD knowledge score + raw SEP score + maternal age + newcomer status + number of previous births. The variance appears approximately constant, so no data transformations or additional analyses were deemed necessary.
Table S4. Supporting information.
