# Supplementary material for: Knowledge about the Developmental Origins of Health and Disease is independently associated with variation in diet quality during pregnancy
Source: Matern Child Nutr. 2019 Dec 12;16(2):e12891. doi: 10.1111/mcn.12891 (PMC7083466; doi:10.1111/mcn.12891)
Supplement: Supplementary file 1 — Figure S1: Comparison of plots of residual error versus fitted values between analysis 2 model using raw SEP score (DOHaDKNOWLEDGE score~raw SEP score + maternal age + newcomer status + number of previous births) and sensitivity model using transformed SEP score (DOHaDKNOWLEDGE score~ (SEP score)5 + maternal age + newcomer status + number of previous births). Figure S2: Residual error versus fitted values from Diet Quality score~ DOHaD knowledge score + raw SEP score + maternal age + newcomer status + number of previous births. The variance appears approximately constant, so no data transformations or additional analyses were deemed necessary. Table S4. Supporting information. [file MCN-16-e12891-s001.docx]

**S Text 1: Brief description of responses to open-ended questions about recommended foods to eat and those to avoid during pregnancy**

Two hundred seventy participants responded to the open-ended, qualitative question about what foods pregnant people should avoid to reduce health risks to themselves and their babies. Two hundred forty-eight (92%) of these responses appeared to be in line with recommendations and resources related to healthy diet during pregnancy from Health Canada, with most respondents reporting that one or more of the following foods should be avoided: raw or undercooked animal products (including meat, eggs, fish); high trophic level fish (e.g. albacore tuna); unpasteurized food and drink (especially soft or ‘blue’ cheeses); processed meat (e.g. hot dogs and deli meats); sugar-sweetened beverages (e.g. soda).

Two hundred forty-two participants also provided responses to the open-ended question regarding what should be eaten during pregnancy. Only five (2%) of the responses to this question appeared to be in contradiction to Health Canada’s guidelines, although many of the responses to this question were not specific enough to code as being in or out of keeping with the formal recommendations (e.g. “lots of healthy foods”, “whole foods”).

In sum, the open-ended responses are generally in line with pregnancy dietary/ health recommendations outlined by Health Canada, further supporting the view that respondents are generally very familiar with the guidelines, and with what experts recommend in terms of pregnancy diet.

**S Text 2: Complete list of all dietary items included in the PrimeScreen Food Frequency Questionnaire**

To calculate respondents’ pregnancy Diet Quality scores, we used the PrimeScreen Food Frequency questionnaire embedded in our pregnancy health questionnaire. The main text provides examples of the kinds of food categories covered in the PrimeScreen tool, and how responses about reported frequency of consumption of foods from various categories were scored. S Table 3 below reports a full list of all 20 food categories in the tool:

S Table 1: Full list of 20 food categories used in food frequency assessment tool:

|  | More than once/ day | Almost daily | 2-4 times/ week | Once/ week | Less than once/ week | Never |
| --- | --- | --- | --- | --- | --- | --- |
| **Broccoli, rapini, cauliflower, cabbage, brussel sprouts** | 4 | 3 | 2 | 1 | 0 | 0 |
| **Carrots** | 4 | 3 | 2 | 1 | 0 | 0 |
| **Other vegetables such as peas, corn, green beans,**  **tomatoes, squash** | 4 | 3 | 2 | 1 | 0 | 0 |
| **Beans, split peas or lentils** | 4 | 3 | 2 | 1 | 0 | 0 |
| **Dark green leafy vegetables such as spinach, romaine lettuce, kale, turnip greens, bokchoy, swiss chard** | 4 | 3 | 2 | 1 | 0 | 0 |
| **Citrus fruits such as oranges, grapefruits** | 4 | 3 | 2 | 1 | 0 | 0 |
| **Other fruits such as fresh apples or pears, bananas, berries, grapes, melons** | 4 | 3 | 2 | 1 | 0 | 0 |
| **Whole milk dairy foods such as homogenized/3.2% milk, hard cheese, butter, ice cream** | -4 | -3 | -2 | -1 | 0 | 0 |
| **Low-fat milk or alternative products such as skim/1%, 2% milk, yogurt, cottage cheese** | 8 | 6 | 4 | 2 | 0 | 0 |
| **Whole eggs** | 2 | 4 | 2 | 1 | 0 | 0 |
| **Beef, pork or lamb** | -4 | -3 | -2 | -1 | 0 | 0 |
| **Turkey or chicken** | 4 | 3 | 2 | 1 | 0 | 0 |
| **Fish/seafood** | 4 | 3 | 2 | 1 | 0 | 0 |
| **Margarine** | -4 | -3 | -2 | -1 | 0 | 0 |
| **Refined grains such as white bread, white rice, white buns** | -4 | -3 | -2 | -1 | 0 | 0 |
| **Whole grain breads and cereals such as whole wheat, oatmeal, brown rice, barley** | 4 | 3 | 2 | 1 | 0 | 0 |
| **Baked products such as muffins, doughnuts, cookies, cake, pastries** | -4 | -3 | -2 | -1 | 0 | 0 |
| **Sugary drinks such as regular soda, fruit drinks, Nestea, Gatorade** | -4 | -3 | -2 | -1 | 0 | 0 |
| **Deep fried foods such as French fries, fried chicken, fish and chips, spring rolls** | -4 | -3 | -2 | -1 | 0 | 0 |
| **Salt added to food at the table?** | -4 | -3 | -2 | -1 | 0 | 0 |

**S Text 3: Further details on collation of and correlations among socio-demographic variables**

Respondents’ highest completed educational attainment level was an ordinal variable. For the purposes of subsequent analyses, educational attainment fell into four, ranked categories: 1) had not obtained a high school diploma, 2) high school diploma, but no college or university, 3) some college or university, and 4) completed post-secondary degree. Finer-grained measures (six ranked categories) of educational attainment were available for most of the sample, but not for the first 78 respondents, so we binned all responses into categories represented in both an earlier and a later, slightly amended version of the questionnaire. The binning strategy is presented in S Table 2.

S Table 2: Measuring maternal educational attainment level.

What best describes the highest level of education you’ve completed?

|  | Questionnaire version 1 | Questionnaire version 2 | Bin used in analysis |
| --- | --- | --- | --- |
| Up to grade 8 | N/A | 1 | 1 |
| More than grade 8 but did not finish/ have not finished high school | 1 | 2 | 1 |
| Finished high school, no college or university | 2 | 3 | 2 |
| Some college or university | 3 | 4 | 3 |
| College diploma or university degree | 4 | 5 | 4 |
| Some post-degree education (e.g. professional program, graduate school) | N/A | 6 | 4 |

To calculate socioeconomic position (SEP score), we added each respondent’s educational attainment score to her household income score (1=<$23,000/ year, 2=$23,000 to $39,999, 3=$40,000-$79,999, 4=>$80,000). For five participants who provided answers to all other questions related to the quantitative analysis used in this study but who chose not to answer (either by skipping the question or selecting “prefer not to say”) the question “What is your total yearly household income from all sources before taxes?”, we inferred a household income score according to the following decision rules. Those who reported social assistance as a main income source were placed in the lowest bracket (<$23,000); those who reported no reliance on social assistance but did not report any indictors of affluence (e.g. rental income, investment income, inheritance or familial wealth) or advanced education (university degree, advanced degree) were placed in the second highest bracket (i.e. the one that includes the city’s median household income for two parent households; $40,000-79,000); those who reported no reliance on social assistance and also reported any other indicators of affluence were placed in the highest bracket (>$80,000). An explanation of each SEP score, ranging from two to eight, is presented in S Table 2. There were no cases in which participants without any post-secondary came from households in the top income bracket, so these scoring combinations are not listed.

S Table 3: Calculation of SEP scores, including all possible values from two to eight.

| SEP Score | Description of SEP score. |
| --- | --- |
| 2 | Participant had not completed high school and had a household income of less than $23,000/ year. |
| 3 | Participant had not completed high school and had a household income of between $23,000 and $39,999 OR participant had completed high school and had a household income of less than $23,000. |
| 4 | Participant had not completed high school and had a household income of between $40,000 and 79,999 OR participant had completed highschool and had a household income between $23,000 and $39,999 OR participant had completed some post-secondary and had a household income below $23,000. |
| 5 | Participant had completed high school and had a household income between $40,000 and $79,000 OR participant had completed some post-secondary and had a household income between $23,000 and $39,000 OR participant had completed a college diploma or university degree and had a household income of less than $23,000. |
| 6 | Participant had completed some college or university and had a household income of between $40,000 and $79,000 OR participant had a college diploma or university degree and had household income between $23,000 and 39,000. |
| 7 | Participant had completed some college or university and had a household income of >$80,000 OR participant had a college diploma or university degree and had household income between $40,000 and $79,000. |
| 8 | Participant had a college diploma or university degree and had household income >$80,000. |

In addition to the variables used to calculate SEP score, we had access to the following socio-demographic characteristics: status as a newcomer to Canada, number of previous births, marital status, self-identified ethnicity. In particular, there were highly significant differences in mean SEP score between the group of respondents who were in married/common relationships (6.8) and those who were not (4.0, p<0.000). Similarly, respondents who reported being newcomers to Canada were disproportionately likely to identify ethnically as being from any cultural group other than white/European/Euro-Canadian (X-squared = 44.9, p<0.000).

We opted to focus on SEP score rather than marital status in our models because SEP is an established and consistent predictor of both health literacy and general self-efficacy whereas this is not always the case with marital status. In fact, from our qualitative interviews and stakeholder meetings, single mothers in the study population were relatively likely to feel they had more control and understanding over their lives, their children’s lives, and their health and nutrition than did women in stable domestic partnerships (unpublished data).

With respect to racialization/self-reported ethnicity versus immigration/newcomer status, Canadian data generally show that linguistic and cultural barriers and especially lack of familiarity with the health system present the most important obstacles to health literacy and self-efficacy, although this may in part the result of research bias in Canada, which has focused largely on immigration status and ignored racialization (Khan et al. 2015). Given this possible research bias, we here present also a supplementary model in which we adjust for white (scored as 0) versus non-white (scored as 1) self-reported ethnicity (S Table 4)

| Variable | Estimate | standard error | p-value |
| --- | --- | --- | --- |
| DOHaD_KNOWLEDGE_ Score | 0.29 | 0.12 | 0.012 |
| Pregnancy Guideline_KNOWLEDGE_ Score | 0.12 | 0.24 | 0.615 |
| SEP score | 0.73 | 0.33 | 0.030 |
| Maternal age | 0.30 | 0.11 | 0.005 |
| Number of previous births | -1.28 | 0.47 | 0.007 |
| Self-reported ethnicity, non-white=1 | -1.00 | 1.17 | 0.391 |

**S Text 4: Exploration of regression residuals, and sensitivity analysis**

Our second and third analyses are both linear mixed effects regression analyses. The diagnostic plots of residual error versus fitted values suggest that the variance in the error for the model used in the second analysis is non-constant, with variance increasing as values of x increase (S Figure 1a). Some data exploration suggests that this heteroscedasticity can be attributed to the massive over-representation of respondents with SEP scores of eight. Although we did not perform a formal Box-Cox analysis, we explored various power transformations of SEP score, raising it to the second, third, fourth, and finally the fifth power. Only raising it to the fifth power appeared to normalize the residuals in the model used in our second analysis (S Figure 1b). The variance of the residuals versus fitted values from the model used in our third analysis appears roughly constant (S Figure 2).

S Figure 1: Comparison of plots of residual error versus fitted values between analysis 2 model using raw SEP score (DOHaD_KNOWLEDGE_ score~raw SEP score + maternal age + newcomer status + number of previous births) and sensitivity model using transformed SEP score (DOHaD_KNOWLEDGE_ score~ (SEP score)^5^ + maternal age + newcomer status + number of previous births).

| 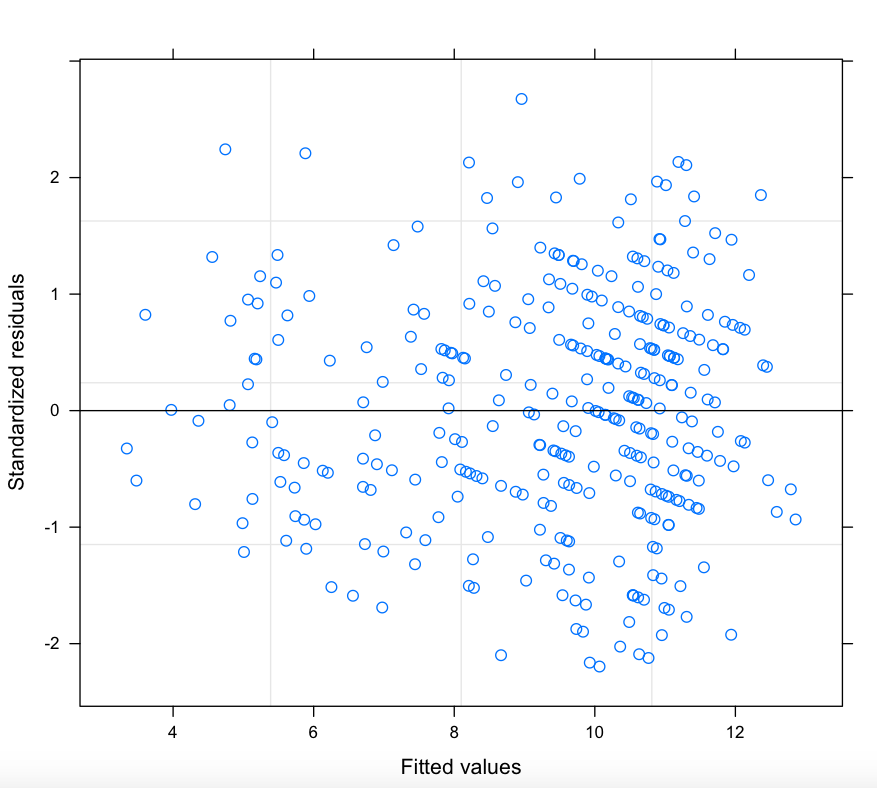 | 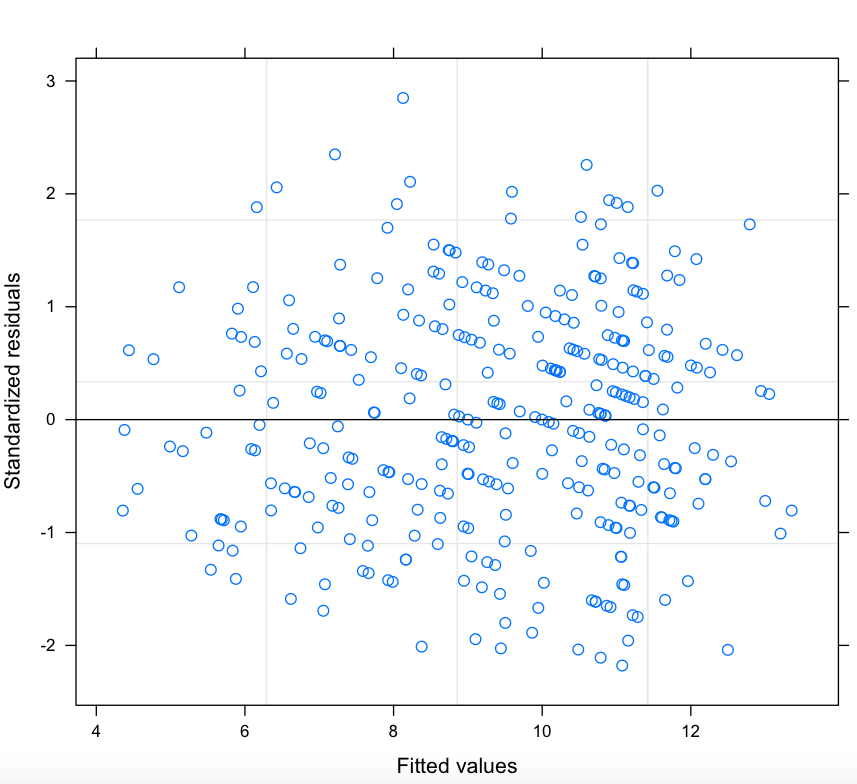 |
| --- | --- |
| S Fig 1a. Residuals versus fitted values from model including raw SEP score as fixed effect. | S Fig 1b. Residuals versus fitted values from model including transformed (exp 5) SEP score as fixed effect. |

S Figure 2: Residual error versus fitted values from Diet Quality score~ DOHaD knowledge score + raw SEP score + maternal age + newcomer status + number of previous births. The variance appears approximately constant, so no data transformations or additional analyses were deemed necessary.

| 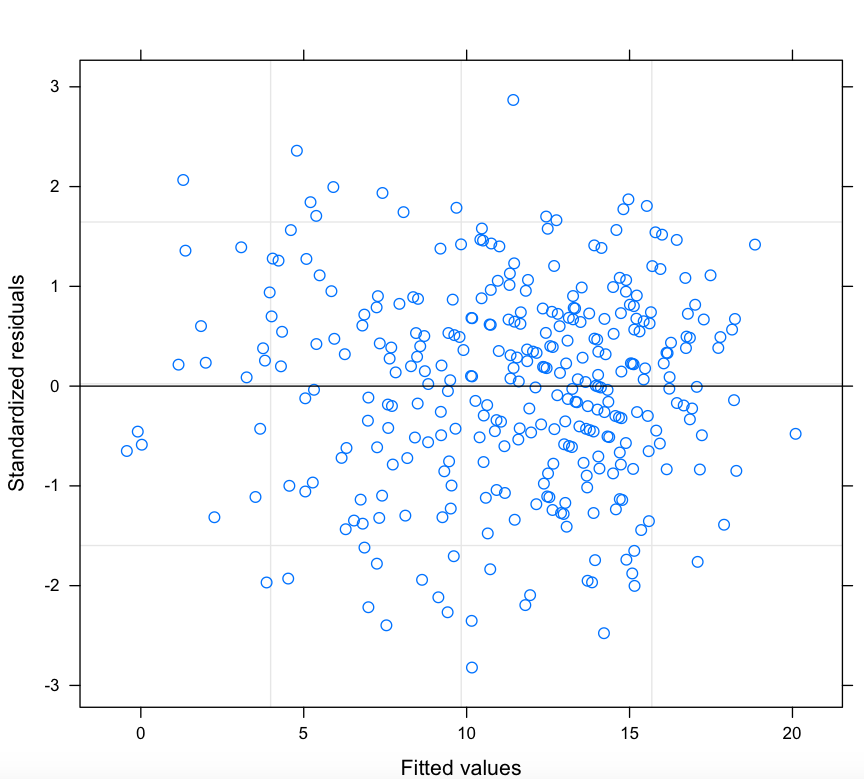 |
| --- |

The unevenness of the variance in the residual error of the model used in our second analysis is problematic, as this is in violation of the assumptions of linear regression analyses, meaning the error terms and p-values generated by this model are likely mis-specified. We nonetheless chose to report the model using the raw values of SEP score in the main text of our paper, largely because relationships between untransformed variables are relatively easier to interpret than are relationships between transformed ones. That being said, we also report sensitivity analyses here in which we use SEP score raised to the power of five as a fixed effect in a model otherwise identical to our main analysis. We compare the estimates, error, and p-values in the model with the raw value with those from the transformed value, and show that they are very similar and that our main results and conclusions hold if we use the transformed value (S Table 4).

S Table 4

|  | Main analysis using raw SEP score | | | Sensitivity analysis using transformed SEP score | | |
| --- | --- | --- | --- | --- | --- | --- |
|  | Estimate | Standard Error | p-value | Estimate | Standard Error | p-value |
| SEP score | 0.78 | 0.15 | 0.000 | 0.00 | 0.00 | 0.000 |
| Maternal age | 0.12 | 0.05 | 0.021 | 1.15 | 0.05 | 0.003 |
| Number of previous births | -0.47 | 0.23 | 0.041 | -0.62 | 0.22 | 0.006 |
| Newcomer status | 1.44 | 0.82 | 0.078 | 1.55 | 0.83 | 0.064 |
| Pregnancy Guideline_KNOWLEDGE_ | 0.22 | 0.12 | 0.064 | 0.25 | 0.12 | 0.037 |

**References:**

Khan, M, Kobayashi, K, Lee, SM, Veng, Z (2015) (In)visible minorities in Canadian health data and research. *Discussion Paper Series: Population Change and Lifecourse Strategic Knowledge Cluster*, 3 (5): 1-33.
